# On Egg-Like Incrustations in Urine

**Published:** 1813-12

**Authors:** G. H. Toulmin

**Affiliations:** Wolverhampton


*To the Editor of the Medical and Physical Journal.*

SIR,

I ATTENDED some time ago, in my professional capacity as physician, a person laboring under diseased liver and dropsy, whose urine, while unusually thick and turbid, much abounded with bile.

---

On

On coming into the sick chamber one morning, the nurse, with tokens of much surprise, exclaimed, that a most extraordinary circumstance had happened! When, pointing to the urine, there appeared floating on its surface a number of bodies of different magnitudes, which had the appearance of light-brown colored, dark-spotted, small birds-eggs.

I carefully took one, about three-fourths of an inch in length, and relatively proportioned, into the palm of my hand, and looked attentively at it for some time. On a slight pressure, it shivered into small thin pieces, devoid of apparent contents.

This is a phenomenon that doubtless must have been noticed by others, but which I never saw before, though I have been more or less extensively engaged in the practice of medicine for a long period.

It would seem easily accounted for, by conceiving air-bubbles to have formed on the surface of the urine, and to have obtained the shape of eggs, by being encrusted with phosphate of lime, or the composition productive of egg-shells, and which doubtless abounded in the fluid.

These bodies, which, in external appearance, perfectly resembled birds-eggs, show that there is a natural determination in the component parts of the species of testaceous matter, of which both are constituted, whether as enveloping air or still more solid materials, under particular circumstances, to assume the egg-like form.

I mention the facts as they occurred to me; and as they seem to lead to conclusions, in regard to the shape and exterior formation of eggs, not generally admitted, I could wish you to let them have a place in your valuable Journal.

*Wolverhampton, Sept.* 7, 1813. G. H. TOULMIN.

